# Additional chromosomal abnormalities in Philadelphia positive chronic myeloid leukemia

**DOI:** 10.12669/pjms.36.2.1384

**Published:** 2020

**Authors:** Sunila Tashfeen, Rafia Mahmood, Saleem Ahmed Khan, Tahir Khadim

**Affiliations:** 1 Dr. Sunila Tashfeen, FCPS. Department of Pathology, Combined Military Hospital, Quetta, Pakistan; 2 Dr. Rafia Mahmood, FCPS. Armed Forces Institute of Pathology, Rawalpindi, Pakistan; 3 Dr. Saleem Ahmed Khan, FCPS, Ph D Haematology, Army Medical College, Rawalpindi, Pakistan; 4 Dr. Tahir Khadim, FCPS. Armed Forces Institute of Pathology, Rawalpindi, Pakistan

**Keywords:** Chronic myeloid leukemia, Conventional cytogenetics, Philadelphia chromosome

## Abstract

**Objective:**

To determine the frequency of additional chromosomal abnormalities in Philadelphia chromosome positive Chronic Myeloid Leukemia (CML) by conventional cytogenetic analysis.

**Methods:**

This descriptive cross sectional study was conducted at Armed Forces Institute of Pathology (AFIP), Rawalpindi, from January 2012 to December 2016. A total number of 528 newly diagnosed CML patients were included in the study. The subjects were tested for the presence of Philadelphia (Ph) chromosome and other additional cytogenetic abnormalities by conventional cytogenetic analysis interpreted according to International System of Human Cytogenetic Nomenclature (ISCN) criteria. Molecular analysis for BCR-ABL was also performed for each patient. The additional cytogenetic abnormalities were then classified into major route abnormalities and minor route abnormalities.

**Results:**

Out of the 528 newly diagnosed CML patients, 378 (71.6%) were males and 150 (28.4%) were females. The age of patients ranged between 18 to 74 years. Four hundred and ninety-eight (94.3%) patients showed Philadelphia chromosome on karyotyping while 30 (5.7%) were negative for the Philadelphia chromosome. On analysis of these 498 Philadelphia positive patients, additional cytogenetic aberrations were detected in 26 (4.9%) patients. Of these, 7 (1.3%) had major route abnormalities while 19 (3.6%) had minor route abnormalities.

**Conclusion:**

The frequency of additional chromosomal abnormalities in our study were not in accordance with previous local and international studies.

## INTRODUCTION

Chronic myeloid leukemia (CML) is a clonal myeloproliferative neoplasm (MPN), characterised by increased proliferation of myeloid cells demonstrating a bimodal peak of myelocytes and neutrophils in peripheral blood and bone marrow.^1^ It is one of the most common haematological malignancies in Asia that involve 15 to 20% of all adult leukemias.^2^ Annual incidence worldwide is 1-2 cases per 100,000 population.^3^ The natural history of CML involves three phases from chronic phase to an accelerated phase and then finally blast transformation.^2^,^3^

CML is characterised by a reciprocal translocation t(9;22) resulting in the Philadelphia chromosome.^4^ This hallmark genetic alteration in CML is essential for diagnosis of this chronic myeloproliferative neoplasm.^5^ This t(9;22) resulting in BCR-ABL fusion gene is detected by RT-PCR or fluorescent in situ hybridisation on molecular analysis while the resulting Ph Chromosome can be detected on conventional cytogenetics.^6^ Thus, these not only are necessary for establishing diagnosis but also have a pivotal role in disease monitoring, thus allowing clinicians to make treatment decisions^7^

Over the years, with better understanding of disease, we do now know that in 5-10% of the patients, BCR-ABL fusion gene is detected on molecular analysis but there is no Philadelphia chromosome.^8^ This is because BCR-ABL fusion gene may result from cryptic chromosomal rearrangements, variant and complex translocations.^9^

Cytogenetic aberrations in addition to the Philadelphia chromosome are seen to be associated with a poor prognosis generally.^10^ These additional cytogenetic abnormalities are associated with disease progression.^2^ CML is the first malignancy to be associated with specific cytogenetic and molecular abnormality and progress of CML from chronic phase to accelerated and blast crisis is at many times associated with cytogenetic evolution.^11^ The frequency of additional cytogenetic alterations increases from less than 5% in chronic phase to 30% in accelerated and up to 80% in blast crisis.^12^

Clinical impact of cytogenetic evolution in CML is studied in many studies in many parts of the world. Similarly pattern of genetic instability is also studied in various studies to see that pattern is repetitive or not.^13^ Molecular and cytogenetic evaluation of patients suspected to have CML is of utmost importance in diagnosis and disease monitoring.^14^ Cytogenetic analysis may reveal additional chromosomal abnormalities which may have prognostic significance and may guide treatment decisions.^15^ However, in developing countries patients do not have access to molecular and cytogenetic diagnostic facilities. Thus, there is no data available on additional cytogenetic aberrations in our population. As Armed Forces Institute of Pathology is a tertiary care referral centre, we conducted this study to determine the frequency and prevalence of these extra cytogenetic aberrations in our population of Philadelphia positive CML in order to risk stratify our patients.

## METHODS

This cross-sectional study was conducted at the Department of Haematology, Armed Forces Institute of Pathology, Rawalpindi. The study was conducted from January 2012 to December 2016. Patients aged more than 18 years, both genders, diagnosed as having chronic myeloid leukaemia based on WHO criteria were included in our study. Patients on any treatment were excluded from the study.

Detailed history, physical examination and baseline laboratory investigations were done. Blood counts were performed and peripheral film was examined. Bone marrow aspiration and trephine biopsy was performed. LAP score was assessed. PCR for BCR-ABL and conventional cytogenetics for Philadelphia chromosome were done. All patients who failed to yield adequate growth on cytogenetics were excluded from the study.

RT-PCR for BCR-ABL fusion gene was performed on ABI 7500 RT-PCR analyzer. Cytogenetic analysis was performed by using conventional G banding technique. After patient reassurance and consent, 2-3 ml of venous blood was drawn from the ante cubital vein by aseptic technique and was taken in heparin for cytogenetics. After that, 0.5 ml of heparinized blood was mixed with 7 ml of RPMI 1640 culture medium in culture vessel and then incubated at 37° C for 72 hrs. 0.2 ml of colchicine was added to medium to arrest metaphases and fixed by adding fixative that is 3:1 part absolute methanol/glacial acetic acid. Slides were prepared from cell pellet and stained with leishman stain. At least 20 metaphases were examined by cytovision semi-automated image analysis system and results reported according to the International System for Human Cytogenetic Nomenclature (ISCN).

FISH studies were performed on all samples negative for BCR-ABL by RT-PCR and negative for Philadelphia chromosome on routine cytogenetics. For Interphase FISH analysis, specimens were processed by standard methods. Metasystems BCR-ABL1 dual colour dual fusion probe was applied to the target on the slide. A total of 500 nuclei were analysed per probe set on completely automated Metasystems analysis system by using a fluorescent Zeiss microscope using an orange green spectrum filter.

The additional cytogenetic abnormalities were then classified into major route abnormalities (second Ph chromosome, trisomy 8, isochromosome 17q, trisomy 19) and minor route abnormalities.

All the collected data was entered in statistical package for Social Sciences (SPSS) version 17. The analysed variables included numerical data like age and qualitative data like gender. Results for Ph chromosome in cytogenetic analysis and presence of additional cytogenetic aberrations were noted and analysed.

This study was approved by the Ethical Review Committee (Dated: 16 November 2017) of Armed Forces Institute of Pathology, Rawalpindi. Informed written consent was taken from the patients.

## RESULTS

A total of 528 newly diagnosed CML patients were enrolled in the study. Out of the 528 CML patients, 378 were males (71.6%) and 150 were females (28.4%). Male to female ratio is 2.5:1. The age of patients ranged between 18 to 74 years. Mean age of the CML patients was 39±14 years. On morphological examination, 453 (85.8%) were in chronic phase while 43 (8.1%) were in accelerated phase and 32 (6.1%) were in blast phase. Molecular analysis revealed BCR-ABL1 p210 fusion protein in 499 (94.5%) while 7 (1.3%) showed BCR-ABL1 p190 protein. No patients showed both fusion genes while 22 (4.2%) were undetermined for the type of fusion gene.

Cytogenetic analysis was performed and results were interpreted according to the International System of Human Cytogenetic Nomenclature (ISCN). Four hundred and ninety-eight (94.3%) patients showed Philadelphia chromosome on karyotyping while 30 (5.7%) were negative for the Philadelphia chromosome. FISH analysis showed typical BCR-ABL fusion pattern in 502 (95.1%) patients while atypical BCR-ABL fusion signals were seen in 25 (4.7%) patients while 1 (0.2%) patient showed normal pattern on FISH (no BCR-ABL fusion signal).

**Fig.1:**
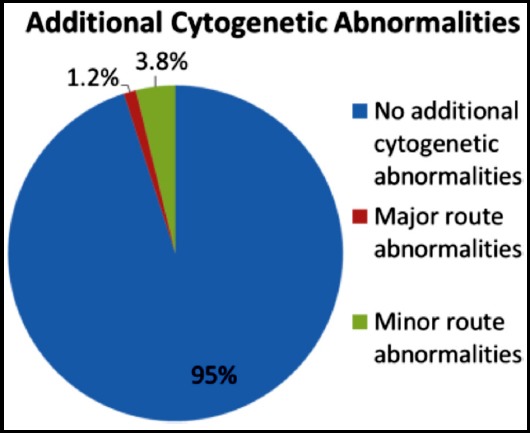
Additional cytogenetic abnormalities.

**Fig.2:**
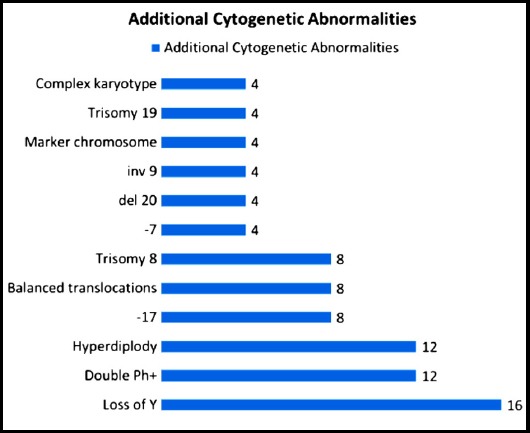
The most common cytogenetic abnormalities.

These patients were further studied on cytogenetic analysis in order to assess additional cytogenetic aberrations. On analysis of these 498 Philadelphia positive patients, additional cytogenetic aberrations were detected in 26 (4.9%) patients. Of these, 7 (1.3%) had major route abnormalities while 19 (3.6%) had minor route abnormalities (Fig.1). The most common cytogenetic aberrations were double Philadelphia and loss of Y (Fig.2).

## DISCUSSION

Myeloproliferative neoplasms (MPN) are heterogeneous group of disorders arising from transformation in a haemopoietic stem cell and characterised by proliferation of one or more cell lines such as granulocytes, platelets or erythroid cells.^8^ Traditionally MPN have been classified on the basis of Philadelphia chromosome presence.^9^,^10^ Philadelphia positive MPN include chronic myeloid leukaemia which has molecular lesion, the BCR-ABL fusion gene, resulting from Philadelphia translocation.^11^

CML is the commonest adult leukaemia in Asia and can be life threatening once progresses to advance phases.^12^ Severance of CML from myeloid hyperplasia due to other primary and secondary haematological disorders has remained to be an intricate job in certain number of cases.^13^ This disease demonstrates varied clinical presentation and disease evolution which has prognostic implications. Some studies have been conducted to show the relationship between this varied presentation and heterogeneity at cytogenetic and molecular level. But still the role of additional chromosomal abnormalities at diagnosis is in doubt.^14^

The principal observation of our study was the results of cytogenetics. The results of our study are not in accordance with the results of local and international studies. Results of cytogenetic analysis in our study were interpreted according to the International system of Human Cytogenetic Nomenclature (ISCN). Total 498 Philadelphia positive CML patients were included in the study and 25 (5.0%) patients showed additional cytogenetic abnormalities along with Philadelphia chromosome. Common abnormalities observed in our study were loss of Y chromosome, double Philadelphia and hyperdiploidy.

Naveen et al did a similar study in Karachi, Pakistan in year 2008 and had dissimilar results to our study. Two hundred and nineteen Philadelphia positive CML patients were included in their study. Thirty-four (15.5%) out of 219 were found to have additional chromosomal abnormalities. The most frequent abnormality observed in this study was Trisomy 8.^15^ In 2011, Hui-Hua H et al has done a study about additional cytogenetic abnormalities in CML and found that 9 (10%) out of 84 newly diagnosed patients of CML has extra cytogenetic aberrations. In this study double Philadelphia was the most common additional cytogenetic abnormality.^2^

The results of our study and comparing them with other local and international studies suggest that extra cytogenetic abnormalities in Philadelphia positive CML follow a nonrandom pattern. It has been suggested by European Leukemia Net that the presence of Additional cytogenetic abnormalities at diagnosis is a warning sign.^16^ Additional cytogenetic abnormalities are acquired in 60-80% in advance phases of CML and hyperdiploidy has been reported in many case reports of progressed CML.^17^ Furthermore clonal cytogenetic evolution has been considered one of the contributing factor and criteria for advancement in CML disease stage.^18^ Thus, knowledge of the additional cytogenetic abnormalities in our CML patients can be useful in determining prognosis and taking treatment decisions. The mechanism by which these cytogenetic evolution confer their impact is poorly understood and further follow up of these patients is required to see their response to standard tyrosine kinase therapy. The ultimate goal of all diagnostic tests is to lead to improvement in the health care facilities for patients.

## CONCLUSION

In conclusion, today’s world treatments are targeting the disease at the genetic level and more studies are need of the day to provide better comprehension of disease biology, best possible diagnostic facilities and targeted therapies. Moreover, vigilant monitoring of patients is needed to decide if and when a treatment should be alter. It should be discern that all the data, clinical and biological should be collected, examine and interpreted in an accurate and timely manner, for the wellbeing of the present and subsequent patients. Finally, the frequency of additional chromosomal abnormalities in our study were heterogenous to that in previous local and international studies.

### Authors Contribution:

**ST:** Conceived and designed, data collection and analysis, manuscript writing and literature research, is responsible for integrity of research.

**RM:** Manuscript revision, data analysis and interpretation.

**SAK:** Supervised study and conducted analysis.

**TK:** Final manuscript revision.
